# Successful Isolation of Infectious and High Titer Human Monocyte-Derived HIV-1 from Two Subjects with Discontinued Therapy

**DOI:** 10.1371/journal.pone.0065071

**Published:** 2013-05-31

**Authors:** Tong Wang, Younong Xu, Haiying Zhu, Thomas Andrus, Sergei B. Ivanov, Charlotte Pan, Jazel Dolores, Gregory C. Dann, Michael Zhou, Dominic Forte, Zihuan Yang, Sarah Holte, Lawrence Corey, Tuofu Zhu

**Affiliations:** 1 Department of Laboratory Medicine, University of Washington, Seattle, Washington, United States of America; 2 Department of Biostatistics, University of Washington, Seattle, Washington, United States of America; 3 Division of Public Health Sciences, Fred Hutchinson Cancer Research Center, Seattle, Washington, United States of America; 4 Division of Vaccine and Infectious Disease Division, Fred Hutchinson Cancer Research Center, Seattle, Washington, United States of America; 5 Key Laboratory of Functional Protein Research of Guangdong Higher Education Institutes, Guangzhou, Guangdong, China; 6 Institute of Life and Health Engineering, College of Life Science and Technology, Jinan University, Guangzhou, Guangdong, China; Institut National de la Santé et de la Recherche Médicale, France

## Abstract

**Background:**

HIV-1 DNA in blood monocytes is considered a viral source of various HIV-1 infected tissue macrophages, which is also known as “Trojan horse” hypothesis. However, whether these DNA can produce virions has been an open question for years, due to the inability of isolating high titer and infectious HIV-1 directly from monocytes.

**Results:**

In this study, we demonstrated successful isolation of two strains of M-HIV-1 (1690 M and 1175 M) from two out of four study subjects, together with their *in vivo* controls, HIV-1 isolated from CD4+ T-cells (T-HIV-1), 1690 T and 1175 T. All M- and T- HIV-1 isolates were detected CCR5-tropic. Both M- HIV-1 exhibited higher levels of replication in monocyte-derived macrophages (MDM) than the two T- HIV-1. Consistent with our previous reports on the subject 1175 with late infection, compartmentalized *env* C2-V3-C3 sequences were identified between 1175 M and 1175 T. In contrast, 1690 M and 1690 T, which were isolated from subject 1690 with relatively earlier infection, showed homogenous *env* C2-V3-C3 sequences. However, multiple reverse transcriptase (RT) inhibitor resistance-associated variations were detected in the Gag-Pol region of 1690 M, but not of 1690 T. By further measuring HIV DNA intracellular copy numbers post-MDM infection, 1690 M was found to have significantly higher DNA synthesis efficiency than 1690 T in macrophages, indicating a higher RT activity, which was confirmed by AZT inhibitory assays.

**Conclusions:**

These results suggested that the M- and T- HIV-1 are compartmentalized in the two study subjects, respectively. Therefore, we demonstrated that under *in vitro* conditions, HIV-1 infected human monocytes can productively release live viruses while differentiating into macrophages.

## Introduction

Peripheral blood monocytes can enter a variety of tissues across capillary endothelial walls, undergoing differentiation to become tissue-specific resident macrophages, such as microglia and perivascular macrophages in the brain, alveolar macrophages in the lung and Kupffer cells in the liver [1], [2]. Based on these migration and differentiation phenotypes, early investigations implicated HIV-1 infected monocytes, carrying provirus, might be able to differentiate into tissue macrophages and develop HIV-1 productive infection *in situ* [3]–[14]. As such, HIV-1 in blood monocytes is considered as a source of progeny viruses in various tissues.

We and others have shown that HIV-1 DNA in patient monocytes are genetically distinct from those derived from CD4+ T cells [15]–[20]. These DNA may have the potential to be activated and release progeny virions. Evidence include studies from us and others [15]–[18], [20] demonstrating that monocyte/macrophages (M/M) are one of the major sources of HIV-1 *in vivo*, particularly in patients receiving highly active antiretroviral therapy (HAART). This deduction is also supported by the fact that the HIV-1 DNA in circulating monocytes has been identified as one of the mechanisms of HIV-1-associated neurocognitive disturbances (HAND) progression [21] and other M/M associated HIV diseases, such as atherosclerosis (reviewed in [22]). Etiologically, although whether these viral DNA in monocytes could produce virions or not is unknown, consistent replenishment of replicable HIV-1 in the central nervous system (CNS) was proposed to be associated with CNS viral persistence and the neurotoxic host inflammatory responses [23]–[27]. On the other hand, reverse trans-endothelial migration of HIV-1 infected M/M was also reported by employing an *in vitro* blood brain barrier (BBB) model, suggesting the possibility that monocytic trafficking from the brain to the periphery may be partially responsible for the rebound of blood viremia [27].

These processes are also known as the “Trojan horse” hypothesis (reviewed in [28]–[31]). Several laboratory-adapted strains have been employed to substantiate this hypothesis in investigative systems including trans-endothelial and co-cultivation models [27], [32]–[35]. However, the *in vivo* relevance of applying these models to neuroAIDS studies could be improved by overcoming a major technological hindrance that has existed for more than two decades: the inability to isolate primary HIV-1 from monocytes that may grow substantially *in vitro*.

Equally important is that HIV-1 DNA in M/M can persist for long periods without integrating, while producing viral mRNAs and proteins as reported recently [36], [37]. The transcriptional products in this process could be detected by PCR, leading to a conclusion of productive infection, however with no progeny virion release examined. Lambotte *et al* [38] and Sonza *et al* [39] have independently reported the isolation of infection-competent HIV-1 from the monocytes of patients while receiving effective HAART. These HIV-1 isolates were at PCR-detectable levels and no infectivity data were reported [38], [39]. While these studies are significant, the transcription of latent HIV-1 DNA that results in viral mRNA release should be considered [36], [37]. Addressing this question warrants direct evidence that monocytes can release virions during the differentiation process to macrophages. This is also a prerequisite of the “Trojan horse” hypothesis if the monocyte-derived HIV-1 DNA is a substantial source of brain HIV-1.

It is known that activated CD4^+^ T cells are capable of sustaining rapid and exponential HIV-1 production [40]–[42], and latently infected CD4^+^ T cells, including resting and central memory T cells, are viral reservoirs in the peripheral blood [43]–[51]. However, later studies indicated that in patients with suppressive HAART, non-CD4^+^ T lymphocytes, including M/M, are the major sources of plasma HIV-1 [15], [20], [50], [52] (reviewed in [19], [53], [54]). These findings emphasize the possibility that HIV-1 DNA harbored by monocytes may be a source of live viruses responsible for viral rebound post-discontinued therapy, which has not been substantiated to date.

Therefore, we focused on our well-studied subjects [15], [17], [18], [20] in this study and described the successful isolation of monocyte-derived primary HIV-1 (M- HIV-1) and their *in vivo* controls, CD4+ T cell-derived primary HIV-1 (T- HIV-1), from two out of four study subjects. These findings are direct evidence of monocytic release of HIV-1 live viruses, which is favorable to the “Trojan horse” hypothesis in HIV-1 tissue transmission.

## Results

### Isolation of HIV-1 from Purified Patient Monocytes and CD4+ T cells

Peripheral blood mononuclear cell (PBMC) samples of Subjects 1175, 1690, 1696 and 1155 from the Seattle Primary Infection Cohort (SeaPIC) [55], [56], who received discontinued therapy, were used for viral isolation. The scientific and ethics review committees of the University of Washington approved this study, and written informed consents were obtained from the study participants. We complied with the human experimentation guidelines of the US Department of Health and Human Services when obtaining clinical samples.

**Figure 1:**
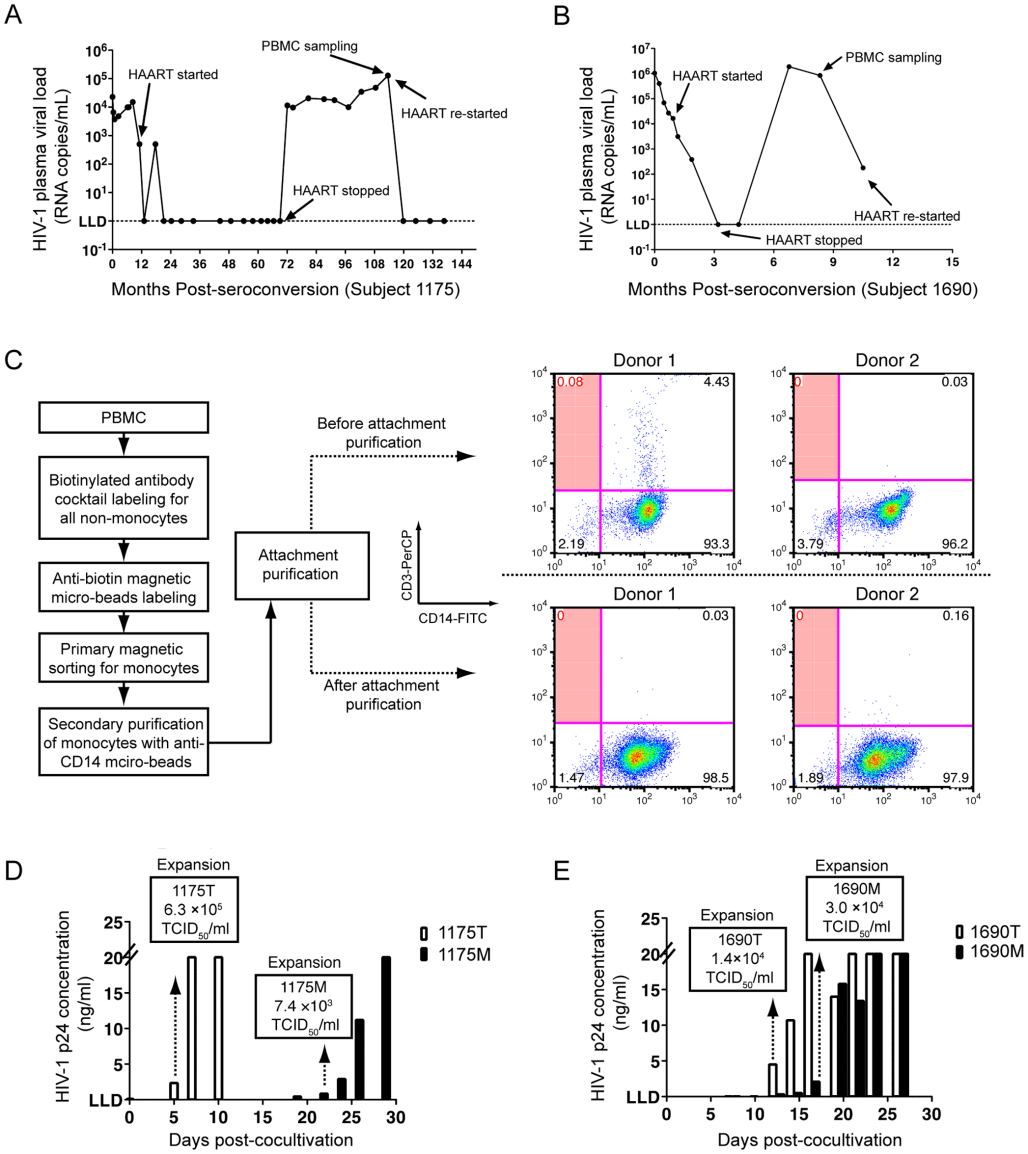
Viral isolation from Subject 1175 and 1690. Clinical information of study Subject 1175 (A) and 1690 (B). For each subject, the first visit with confirmative diagnosis of seroconversion, along with the corresponding HIV-1 RNA viral load, is plotted at Month 0. Subsequently, longitudinal visits, viral loads, treatment and sampling time points are shown. (C) The monocyte purification strategy. Non-monocytes were magnetically labeled and removed from PBMCs of 2 healthy donors. The eluent monocytes were positively sorted and subjected to a four-hour attachment purification followed by two intensive washes with PBS. Percentage of T cell contamination before and after attachment purification was shown in the shaded quadrants of each flow cytometry graph. Monocyte- and CD4+ T cell-derived HIV-1 were obtained from subject 1175 (D) and 1690 (E) by being co-cultivated with CD8+ T cell-depleted donor PBMC. Longitudinal HIV-1 p24 concentrations in supernatants were shown for each co-cultivation. The high limit cut-off of HIV-1 p24 concentration is 20 ng/ml and lower limit for detection (LLD) is 0.01 ng/ml. The time points for viral expansion and stock preparation were indicated with dash lines with arrowheads. TCID_50_ titers of each viral expansion of 1175 T and 1175 M (D), as well as 1690 T and 1690 M (E), are shown in each corresponding box.

As M-HIV-1 isolation was only successful in Subject 1175 and 1690, their treatment information and sampling time points were summarized in Figure 1. In general, the viral loads of both subjects were successfully suppressed to less than 50 copies/ml by HAART, followed by viral load rebound with discontinued therapy. As mentioned in the introduction section, these viruses were rebounded primarily from non-CD4^+^ T cells and most likely derived from monocytes [15]–[18], [20]; this may increase the probability for viral isolation from monocytes. Thus, we acquired PBMC samples for viral isolation at the time points with the longest duration of discontinued therapy for both subjects.

In detail, from 334 to 1001 d post-seroconversion (PSC), subject 1175 was treated with Indinavir, Lamivudine and Zidovudine, followed by a period with no therapy until 3407 d PSC. The subject initiated a second therapy regimen of Efavirenz, Epzicom and Kivexa at 3596 d PSC, the same time point that PBMC samples were acquired for virus isolation (immediately before therapy) (Figure 1A). Subject 1690 was treated with Kaletra and Combivir from 29 d to 166 d PSC, stopped therapy for 188 d, and initiated the same therapy regimen from 336 d PSC (Figure 1B). PBMC samples for 1690 were acquired 295 d PSC (Figure 1B). Plasma viral loads of the two subjects at their respective sampling time points for viral isolation were 1.3×10^5^ copies/ml for 1175 (Figure 1A) and 1.9×10^5^ copies/ml for 1690 (Figure 1B).

We have optimized CD14^+^ monocyte purification by using a two-step, magnetic bead-based cell sorting strategy as previously described [15], [17], [20]. In addition, we included an attachment purification procedure to further remove non-monocyte cell populations. The overall monocyte isolation strategy is shown in Figure 1C.

We validated this strategy with 2 healthy donors by analyzing T cell contamination using flow cytometry (FACS). The feasibility of this validation capitalizes on an interesting feature of monocyte-derived macrophages (MDM) *in vitro* culture. Monocytes will attach to the bottom of the culture surface rapidly (>95% adherence rate at 4 h post seeding), however, over 80% of these attached cells will be released back into culture media 24–48 h post-seeding. Following differentiation, viable MDM will again adhere to the culture surface and remain attached for more than 30 d. This adherence quality allowed us to collect cells after attachment purification for FACS assay at 24 h post-seeding without disturbing cell surface markers. CD3^+^CD14^−^ T cells were grouped in the upper left quadrant of each FACS graph (Figure 1C). For Donor 1, prior to the attachment purification, there was less than 0.1% T cell contamination was detected, however non-detectable T cell contamination was observed after attachment purification (Figure 1C). No T cell contamination was detected either before or after attachment purification in Donor 2 (Figure 1C). We further employed reverse transcription PCR (RT-PCR) to determine T cell receptor (TCR) mRNA levels in the purified monocyte populations, as described [18], and found no detectable TCR mRNA in the purified monocytes of either donors. With this optimized method, we can acquire purified monocytes with no detectable T cell contamination.

We then used this methodology to purify monocytes from all subjects for viral isolation. No detectable TCR mRNA in the purified monocytes of each subject was observed. Out of the four subjects, M-HIV-1 were successfully isolated from two of them (1175 and 1690). HIV-1 primary isolates were defined as either M (monocyte) or T (CD4+ T cell-derived) following the subject ID, that is: 1175 M and 1175 T, or 1690 M and 1690 T represent M- and T- derived HIV-1 isolated from subjects 1175 and 1690, respectively. M- and T- derived HIV-1 were successfully isolated based on p24 ELISA positivity and 50% tissue culture infectious dose (TCID_50_) viral titration (Figure 1D and 1E). By monitoring HIV-1 p24 production in the supernatants, the co-culture for isolating 1175 T first showed p24 ELISA positive on Day 5, while 1175 M was positive on Day 18 (Figure 1D). Comparably, p24 positivity was observed on Day 12 for 1690 T co-culture and Day 13 for 1690 M (Figure 1E). To maximally avoid mutation interference, we collected primary isolates at the first time point of each experiment when p24 production was positively detected. Titers of each viral expansion used for subsequent virological and biological evaluations are indicated by TCID_50_/ml: 1175 T (6.3×10^5^), 1175 M (7.4×10^3^), 1690 T (1.4×10^4^) and 1690 M (3.0×10^4^) TCID_50_/ml (Figure 1D&E).

### Co-receptor Use Prediction and Biological Confirmation

By analyzing the sequences of *env* V3 regions of each HIV-1 isolate, theoretical phenotypes including the syncytium-inducing (SI) effects and co-receptor usage of each isolate were calculated (Table 1.). According to the 11/25 rule [57], 1690 M and 1690 T are non-syncytium-inducing (NSI) viruses; however, 1175 M and 1175 T are SI viruses (Table 1.). In terms of Geno2pheno prediction [58], 1690 M and 1690 T were R5-tropic HIV-1, while 1175 M and 1175 T were predicted as CXCR4 (X4)-tropic viruses (Table 1.). PSSM prediction [59] indicated that 1175 M and 1175 T were CCR5 (R5)-tropic HIV-1, although consistent R5-tropic predictions with Geno2pheno were made for 1690 M and 1690 T (Table 1.). We next biologically evaluated the co-receptor usage of HIV-1 isolates by performing *in vitro* infection experiments with the U87 and U373-MAGI cell lines (Table 1.). Multiple negative and positive controls were used to confirm the validity of the two indicator cell lines as described in the Material and Methods. All of the four isolates could only infect and replicate in U87-CCR5 and U373 MAGI.CCR5 cells, demonstrating R5-tropism (Table 1.).

**Table 1:** HIV-1 Env V3 amino acid sequences with computational and biological prediction of co-receptor usage.

Viruses	Env V3 region amino acid sequences a	11/25rule^b^	Geno2pheno(Subtype)^c^	PSSM(Scores)	U373 celllines	U87 celllines
HXB2	CTRPNNNTRK**R**IRIQRGPGRAFVT**I**GK-IGNMRQAHC					
1690 T	CTRPNNNTRK**S**INI–GPGRAFYA**T**GEIIGDIRQAHC	NSI	R5 (B)	R5 (−8.88)	R5	R5
1690 M	CTRPNNNTRK**S**INI–GPGRAFYA**T**GEIIGDIRQAHC	NSI	R5 (B)	R5 (−8.88)	R5	R5
1175 T	CTRPNNNTRK**G**IHI–GPGRAFYA**R**–IVGDIRQAHC	SI	X4 (B)	R5 (−8.88)	R5	R5
1175 M	CTRPNNNTRK**G**IHI–GPGRAFYA**R**–IVGDIRQAHC	SI	X4 (B)	R5 (−8.88)	R5	R5

a 10 to15 end-point diluted PCR products were directly sequenced from each virus, and the Env V3 regions of 1690 M, 1690 T, 1175 M and 1175 T, aligned to the HXB2 strain, were shown. The positions 11 and 25 of the Env V3 region are indicated in bold. “−” Represents a sequence gap.

b Non-syncytia formation and syncytia formation HIV-1 are abbreviated as NSI and SI, respectively.

c CCR5- and CXCR4- tropic viruses were indicated by R5 and X4, respectively.

### Replication Kinetics in MDM Infection

**Figure 2:**
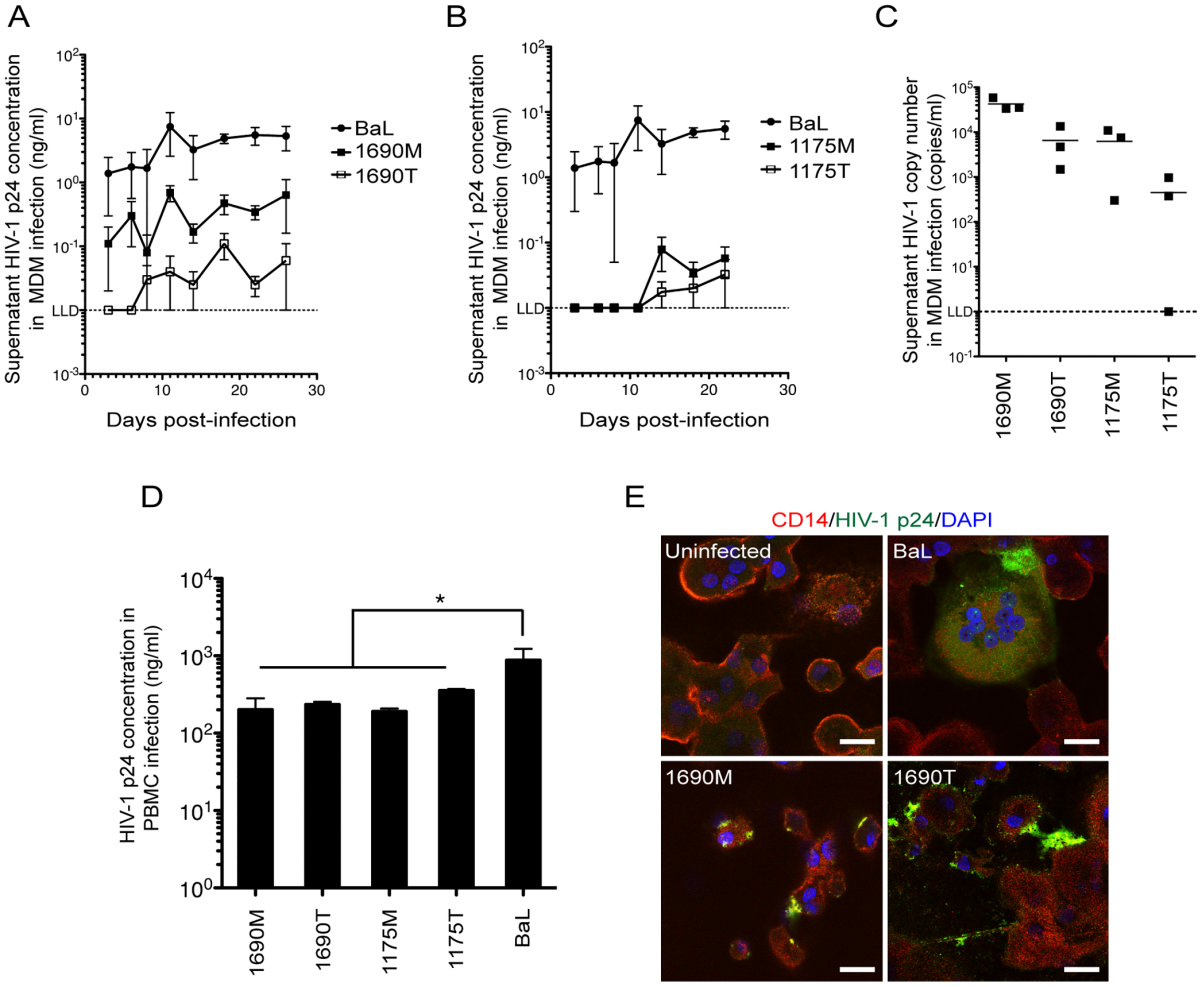
Observation of replication kinetics in MDM. HIV-1 p24 production kinetics in MDM was shown for1690 M (A) and1690 T and 1175 M and 1175 T (B). Seven-day old MDM infection at MOI = 0.01 for 4 h before continuously cultured in fresh media. Supernatant HIV-1 p24 concentrations were determined and shown longitudinally (ng/mL, mean ± SEM, donor n = 4). (C) Supernatant viral RNA copy numbers of MDM infections. Supernatant samples were acquired on Day 22 post-infection and data were shown as copies/mL (donor n = 3). (D) Viral replication in PHA-PBMC. Donor PBMCs were stimulated by PHA for 48 h before BaL, 1690 M, 1690 T, 1175 M and 1175 T infections at MOI = 0.01. HIV-1 p24 concentrations in culture supernatants on Day 7 post-infection were shown (ng/mL, mean ± SEM). *P<0.05, donor n = 4 compared with the BaL-infected group. *E,* Intracellular staining of HIV-1 p24. MDM were infected for 14 d before cyto-immunochemistry staining with CD14 (red), DAPI (blue) and HIV-1 p24 (green). Scale bar = 20 µm.

To validate whether the M- and T- HIV-1 were isolated from different compartments, we compared the phenotypic difference, as the first step, between these isolates by infecting MDM that were prepared from 4 individual healthy donors, respectively; as a positive control, parallel experiments were performed with HIV-1 BaL (Figure 2).

Using a linear model and generalized estimating equations (GEE) with exchangeable correlation structure for repeated measures from the same donor [60], the 1690 M infected group showed significantly higher HIV-1 p24 production than the 1690 T group, longitudinally (*P*<0.01, donor n = 4, Figure 2A). The 1175 M and 1175 T infected groups showed p24 production at levels close to the ELISA lower limit of detection (LLD, 0.01 ng/ml) for most of the time points; and the 1175 M infected MDM showed a general pattern of higher p24 production than the 1175 T infected group (Figure 2B), especially on Day 18 post-infection (*P*<0.05, donor n = 4). The HIV-1 infected MDM were observed to maintain healthy morphologies until Day 22 post-infection, and gradually show cytotoxic morphologies thereafter, such as shrinkage and decrease in cell numbers. As such, to validate the replication difference between M- and T- HIV-1, we used quantitative PCR to examine supernatant viral RNA copy numbers on Day 22 post-infection in the 1690 M, 1690 T, 1175 M and 1175 T infected MDM (Figure 2C). The comparison was performed with linear regression and GEE with exchangeable correlation structure for repeated measures from the same donor, within virus from the same patient. The average viral RNA copy number of the M-HIV-1 infected groups was 1.1×10^4^ copies/ml, which is significantly higher than the T-HIV-1 infected groups (5.8×10^2^ copies/ml) (*P*<0.01, donor n = 3, Figure 2C).

As 1175 M, 1175 T and 1690 T showed only moderate RNA or p24 production in MDM, we need test whether these are defective viruses or not. Thus, infection experiments by using PHA-stimulated PBMCs were performed, and peripheral blood from four different healthy donors were used for this validation (Figure 2D). On Day 7 post-infection, supernatant p24 production was seen in the 1690 T (236±19 ng/mL), 1690 M (201±47 ng/mL), 1175 T (355±17 ng/mL) and 1175 M (190±18 ng/mL) infected groups (Figure 2D). These results demonstrate the four primary isolates are replication-competent viruses.

To visualize the viral replication in MDM, we adopted cyto-immunochemistry to assay the HIV-1 p24 production (Figure 2E). As a negative control, uninfected monocytes from healthy donors were allowed to differentiate for 21 d before observation. High power fields (HPF) of observations indicated multinucleated giant cells expressing both CD14 and HIV-1 p24 were observed in the BaL-, 1690 M- and 1690 T- infected MDM, respectively (Figure 2E). These results verified that supernatant p24 and viral production is due to MDM productive infection.

### Genetic Characterization of M- and T- derived HIV-1 *env* C2-V3-C3 Regions

**Figure 3:**
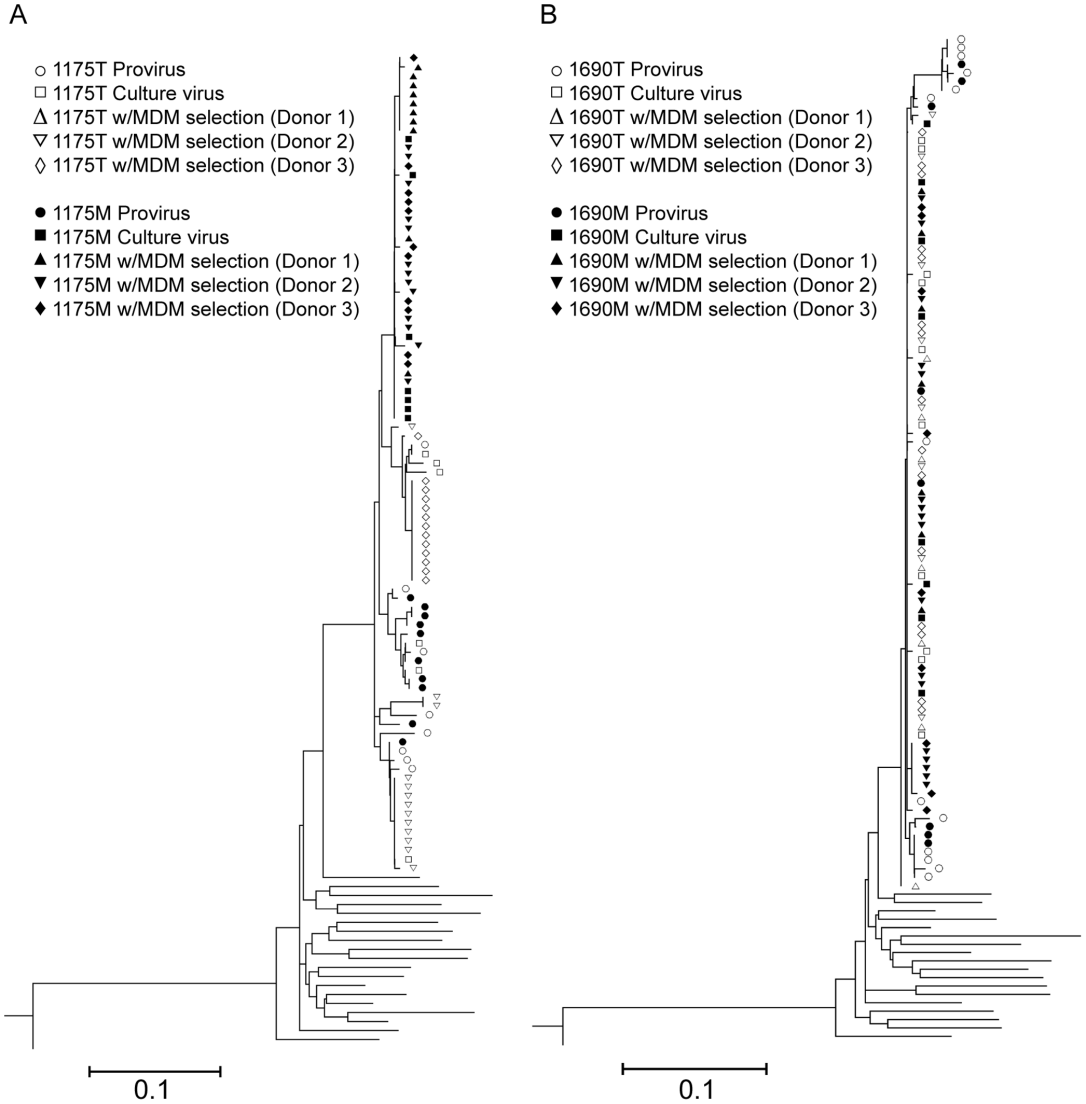
Phylogenetic analysis of HIV-1 *env* C2-V3-C3 sequences of monocyte- and CD4+ T cell-derived HIV-1. The neighbor-joining trees of 1175 M and 1175 T (A) as well as 1690 M and 1690 T (B) were shown, respectively. The *env* C2-V3-C3 sequences of provirues as well as primary isolate before and after MDM selection were acquired from limiting dilution PCR. MDM selection experiments were using seven-day old MDM prepared from 3 healthy donors, respectively, at MOI = 0.01. On Day 22 post-infection, supernatants of infected MDM were subjected to sequence analysis. The viral nucleotide sequences were used to construct neighbor-joining trees based on nucleotide pairwise distances. Reference sequences (not labeled) are HIV-1 subtype B sequences from GenBank database (http://www.ncbi.nlm.nih.gov/).

We next performed HIV-1 sequence analysis on the *env* C2-V3-C3 regions of all of the four primary isolates (1175 M, 1175 T, 1690 M and 1690 T) to detect compartmentaliztion (Figure 3). In addition, to identify the viruses that are replication-competent in macrophages, the MDM selection of the primary M- and T- isolates were used to differentiate the strains that could productively infect MDM (Figure 3). Sequence comparisons with our published proviral sequences [15], [17], [20] were performed to investigate the cell type-specific origin of the isolates (Figure 3). The MDM selection experiments were independently performed for three times using blood M/M from three different healthy donors, respectively (Figure 3).

In order to eliminate re-sampling and PCR-caused errors, we employed our well-established limiting-dilution PCR strategies [15], [17], [20]; each of the end-point diluted PCR products was directly sequenced. We observed identical V3 sequences within limiting-diluted PCR reactions of each isolate. Consistent with our previous reports [15], [17], [20], heterogeneous sequences of HIV-1* env* C2-V3-C3 regions of the proviruses in the monocytes and CD4+ T cells of both 1175 and 1690 were observed (Figure 3A and 3B). Sequence diversities of proviruses from monocytes were 2.08% and 1.79% in 1175 and 1690, respectively. The diversities of proviruses from CD4+ T cells were 3.47% and 2.22% in 1175 and 1690, respectively. There were also notable sequence variations of HIV-1* env* C2-V3-C3 regions from 1175 T (3.48% diversity) and after MDM selection (1.73% in Donor 1 and 1.44% in Donor 2, negative in Donor 3 MDM cultures). On the contrary, before MDM selection, 1175 M showed relatively homogenous sequences (0.8% diversity); however, 1175 M produced more homogenous sequences after MDM selection (0.12% diversity in Donor 1, 0.13% diversity in Donor 2 and 0.06% diversity in Donor 3 MDM cultures) (Figure 3A). Regarding 1175 isolates, we observed only strains with similar sequences to 1175 M could survive the MDM selection. These *env* C2-V3-C3 sequence results indicate 1175 M exists as minor strains of HIV-1 in peripheral blood of subject 1175 and are genetically distinct from 1175 T. The 1175 M strains are capable of replicating in MDM with higher MDM fitness than 1175 T strains from the same subject.

In contrast, homogenous *env* C2-V3-C3 sequences were detected before MDM selection in 1690 M (0.16% diversity) and 1690 T (0.14% diversity), as well as after MDM selection in 1690 M (0.00%,0.15% and 0.35% diversity for Donor1, Donor 2 and Donor 3, respectively) and 1690 T (0.20%, 0.17% and 0.00% diversity for Donor1, Donor 2 and Donor 3, respectively) (Figure 3B).

### Genotypic Differences in the Infectivity-related *gag-pol* Region of MDM Selected 1690 M and 1690 T

**Figure 4:**
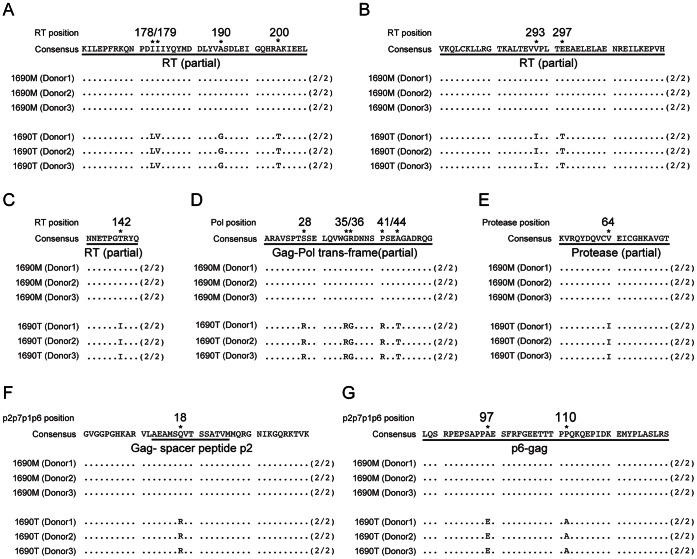
Deduced amino acid sequence alignment of HIV-1 Gag-Pol regions of MDM-selected 1690 M and 1690 T. Amino acid sequence differences between MDM selected 1690 M and 1690 T were shown in the regions of reverse transcriptase (A-C), Gag-Pol trans-frame (D), protease (E), Gag p2 (F) and Gag p6 (G). MDM from 3 different donors (Donor 1, 2 and 3) were infected with 1690 M and 1690 T, respectively. Supernatants were harvested on Day 22 post-infection and subjected to RT-PCR and sequence analysis on Gag-Pol regions of HIV-1. (2/2) represents the same individual sequences were detected from two RT-PCR. Differential residuals were indicated by both “*” and position numbers aligned with HXB2 sequences.

As similar *env* C2-V3-C3 sequences between1690 M and 1690 T were detected, we next examined the *gag-pol* variations to further define the genotypic difference between the two isolates. The consensus sequences of MDM-selected 1690 M and 1690 T were analyzed by directly sequencing PCR products in the supernatants of infected MDM prepared from three healthy donors (Figure 4). The variations after MDM selection between 1690 M and 1690 T shown are consistently detected among all three donors (Figure 4). After MDM selection, a total of seven variations comparing 1690 T with 1690 M were detected in the reverse transcriptase (RT) coding region: L178I, V179I, G190A, T200A, I293V and T297E (Figure 4A&B), as well as I142T (Figure 4C). Five variations were detected in the Gag-Pol trans-frame region: R28S, R35G, G36R, R41P and T44A (Figure 4D). Other observed variations include I64V in the protease region (Figure 4E), R18Q in the Gag-spacer peptide p2 encoding region (Figure 4F), as well as E97A and A110P in the p6 encoding region (Figure 4G).

### Intracellular HIV-1 DNA Production in MDM and RT Activity Difference Comparing 1690 M with 1690 T

**Figure 5:**
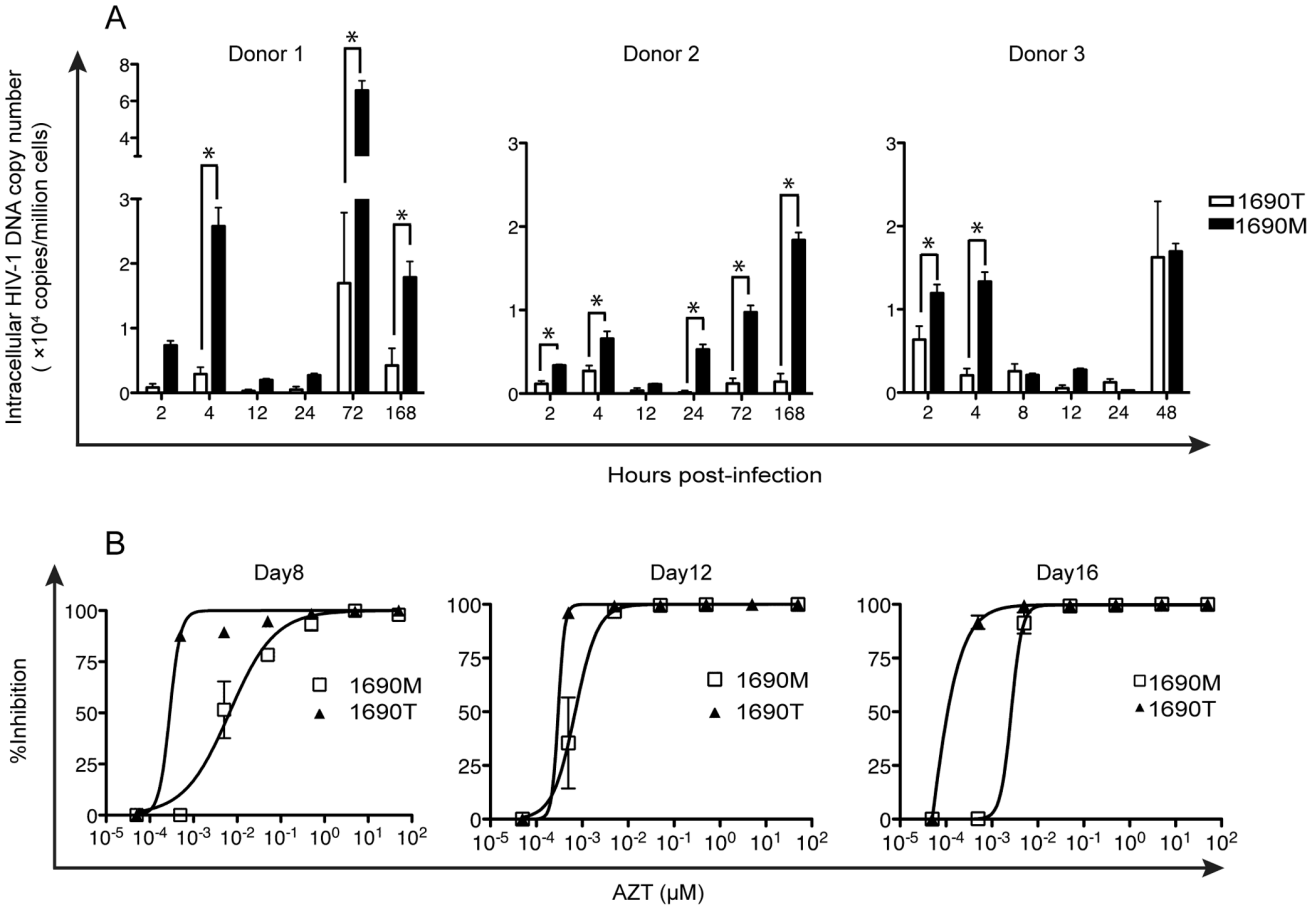
Intracellular viral DNA persistence in MDM acute infection and longitudinal reverse transcriptase activity assay. (A) HIV-1 DNA synthesis kinetics in MDM. Seven-day old MDM from 3 healthy donors were infected by 1690 M and 1690 T, respectively, at MOI = 0.01. Cells were harvested at 2 h, 4 h, 8 h, 12 h, 24 h, 48 h, 72 h and 168 h post-infection. Intracellular HIV-1 DNA copy numbers were determined. *P<0.05, donor n = 3. (B) Viral resistance to azidothymidine. Seven-day old MDM were infected by 1690 M and 1690 T, respectively, at MOI = 0.01. On Day 6 post-infection, supernatants were removed and MDM were exposed to AZT at serially diluted concentrations as indicated. The inhibition rates calculated by relative p24 production were shown for the time points of Day 8, 12 and 16 post-infection, respectively. Data were shown as mean ± SEM, donor n = 3.

Our genetic analyses of 1690 M and 1690 T allowed us to hypothesize that the phenotype difference between the two viruses in MDM is partially due to the RT activity difference. Thus, we further tested the intracellular HIV cDNA production in MDM longitudinally (Figure 5). To calculate intracellular viral cDNA copy numbers in MDM, our published PCR strategy by simultaneously analyzing HIV-1 p24 and p17 cDNA was employed [20], [61]. These experiments were independently repeated for three times, each using MDM prepared from one of the three healthy donors.

Although donor difference was observed, MDM infected with 1690 M showed either significantly higher intracellular DNA copy numbers or no statistical difference from 1690 T-infected MDM, longitudinally (Figure 5A). When evaluating early infection time points, 1690 M showed significantly higher DNA production in MDM than 1690 T at 4 h (*P*<0.01, n = 3), however the absolute amount of intracellular viral DNA decreased from 4–12 h and no significant viral DNA level difference was detected at 12 h post-infection (Figure 5A).

Such significant fluctuations in viral DNA production may be a result of RT activity differences, both in acute and chronic phases post-infection in MDM. In this regard, we performed a longitudinal infection of MDM using 1690 M and 1690 T in the presence of the RT inhibitor, azidothymidine (AZT) (Figure 5B). These experiments were independently repeated three times, each using MDM prepared from one of the three healthy donors. On Day 8 post-infection, the EC_50_ of AZT in blocking RT activity in the 1690 M-infected MDM group was 6.70 nM, significantly higher than the 1690 T group (0.28 nM, *P*<0.01, donor n = 3) (Figure 5B). Similar RT activity differences were detected in the latter time points: 0.70 nM in the 1690 M group and 0.27 nM in the 1690 T group on Day 12 (*P*<0.01, n = 3), as well as 2.7 nM versus 0.32 nM on Day 16 (*P*<0.01, n = 3) (Figure 5B). These results indicate that the RT activity of 1690 M is significantly higher than that of 1690 T.

## Discussion

Currently used HAART regimens fail to effectively inhibit HIV-1 DNA persistence in monocytes, while suppressing HIV-1 in CD4^+^ T cells well [15]–[17], [20], [62]–[65].Our findings in this study pointed toward that these HIV-1 DNA in monocytes are not only transcription-competent, but also capable of producing live viruses along with the monocytic differentiation. This is evidenced by the isolation of M- HIV-1 with both nanogram p24 level and high infectious titers, although only successful in two study subjects. Per these results, we could conclude that the monocytic HIV-1 DNA is an active source of virions in HIV-1 brain transmission. To explain the origin of these viral DNA, Carter *et al* found that the multipotent hematopoietic progenitor cells (HPCs) can be productively infected by HIV-1 [66]. In comparison, recent findings suggested another two types of “Trojan horses”, showing that infected macrophages can migrate inside-out and outside-in the brain [27], and macrophage-derived exosomes and microvesicles are also active vehicles for HIV-1 to mediate productive infection [67]. Taking these together, brain infection of HIV-1 involves in sophisticated mechanisms, but the possibility of viral replenishment via monocytes is confirmed by our findings.

According to the data of subject 1175 we previously reported [17], HIV-1 DNA in monocytes and CD4^+^ T cells had homogenous sequences in the *env* C2-V5 regions during acute infection stages, however significant compartmentalization and variation were found in late stages [17]. This is biologically comparable with our findings here: 1) patient cells utilized for 1175 viral isolation were acquired from late stages of infection, and the proviruses in monocytes and CD4^+^ T cells were known to exhibited compartmentalization [17]; 2) although diversified proviruses were observed in monocytes, homogenous sequences were detected in 1175 M, in contrast to the multiple variants that were isolated in 1175 T. Favorable to our conclusions, there were reports indicating a number of R5 strains found in HIV-1 infected patients cannot enter macrophages at early stages; however, macrophage entry capacity was observed to be increasing longitudinally throughout disease progression [68], [69]. In addition, macrophages are known to express both CCR5 and CXCR4; early investigations by using HIV-1 isolates showed HIV-1 is being adapted to use CXCR4 in addition to CCR5 expressed by both macrophages and CD4^+^ T cells along with infection progression [70], [71]. Adding to this knowledge, our results demonstrated strong monocytic selection on HIV-1 *in vivo*. This is in agreement with the facts that the failure of productive infection of HIV-1 in monocytes is due to post-entry events [72], [73].

Samples for viral isolation were acquired from relatively early infection for Subject 1690; we observed homogenous *env* C2-V3-C3 sequences between 1690 M and 1690 T as anticipated. Therefore, the phenotypic difference between the two isolates could be partially explained by replication-associated variation in viral sequences. We demonstrated that 1690 M has more resistance variation sites than those from CD4^+^ T cells. These sites include the well documented non-nucleoside RT inhibitor (NNRTI) resistance-associated mutation, G190A [74]–[76], when comparing 1690 T with 1690 M, as well as four others that are known to confer lesser degrees of NNRTI resistance, including V179I [77], I293V [78] and T200I [77]. In addition, the I64V mutation in the protease region was also reported to be relevant to protease inhibitor resistance [79]. Indeed, it is known that acquiring these mutations is to be beneficial for HIV-1 by increasing fitness in host cells, representing a monocyte selection mechanism of concentrating viruses that have higher RT activity [39].

This was supported by the results of HIV-1 DNA copy number calculation post-entry. We found the 1690 M-infected MDM showed significantly higher viral DNA production than those in the 1690 T group for the first 4 h post-infection. It has been reported that the minimum duration of an HIV-1 replication cycle *in vivo* is about 1.2 d and the average duplication time is 2.6 d [42]. During this process, reverse transcription requires nearly 4 h in both CD4^+^ T cells and mononuclear phagocytes [42], [80]. However, subsequent events, including integration and progeny viral assembly, are slower in the latter [80]. As DNA production in the 4 h time window is completely dependent on the efficiency of reverse transcription, RT activity difference between 1690 M and 1690 T became a focus of mechanistic probing in this study. Notably, by employing the RT inhibitor AZT, we validated that 1690 M has higher RT activity than 1690 T.

Timely and sufficient reverse transcription is a crucial prerequisite for the survival of HIV-1 in monocytes [81]. It has been demonstrated that HIV-1 post-entry block in monocytes occurs prior to or during the reverse transcription phase [9], [72], [81]. Slower DNA synthesis results in increased DNA degradation, while insufficient reverse transcription leads to integration block and increased levels of unintegrated DNA. Compared with macrophages, there are abundant factors in monocytes that may confer the inhibition of reverse transcription. They include lower levels of dTTP [9], higher levels of APOBEC3A and APOBEC3G [82], [83] as well as the recently identified HIV-1 reverse transcription restriction factor, SAM- and HD-domain-containing protein (SAMHD1) [84], [85]. Therefore, to meet the requirement of the rate and quality of viral DNA production for survival in monocytes, higher RT activity is demanded. This is in agreement with our previous report indicating that after prolonged HAART, HIV-1 variants related or identical to those found only in CD14+ monocytes were also seen in plasma from treated subjects, suggesting that HIV-1 in circulating monocytes represent replicating virus that were able to survive with effective HAART [20].

In conclusion, the above-mentioned genotype and phenotype results confirmed that the M- and T- HIV-1 were isolated from different cellular compartments in the two subjects, respectively. Therefore, we demonstrated that infectious HIV-1 can be recovered from the monocytes of infected patients. These findings are the first biological evidence of monocyte-derived HIV-1, leading to a better understanding of cell-specific viral persistence and the “Trojan horse” hypothesis in HIV-1 CNS transmission. The M-HIV-1 may be applied to neuroAIDS and other HIV-1 disease modeling systems, as well as further benefit the biological and pharmaceutical evaluation of monocyte/macrophage-related HIV-1 pathogenesis.

## Materials and Methods

### Isolation of Infectious HIV-1 from Purified Monocytes

CD14+ monocytes were first purified from PBMC by a negative selection kit (Miltenyi Biotec, Auburn, CA) containing a cocktail of biotinylated anti- CD3, CD7, CD19, CD45RA and CD56 and immunoglobulin E (IgE) antibodies (Abs). By labeling with the primary antibody cocktail and secondary anti-biotin microbeads, non-monocyte populations including T cells, B cells, NK cells, granulocytes, dendritic cells and basophils were magnetized and trapped by the cell-sorting column (Miltenyi). The eluent fraction containing enriched monocytes was labeled with a microbead-conjugated anti-human CD14 Ab (Miltenyi) and purified through an additional positive selection. Monocytes were then allowed to attach to culture surfaces for 4h, followed by culture media washes until no floating cells observed.

The purity of monocytes was then determined by flow cytometry with fluorescent labeling of anti-human CD14, CD3 and CD4 Abs (Miltenyi), and analyzed by a FACSCalibur flow cytometer (Becton Dickinson, San Jose, CA). With this purification method, we can acquire monocytes with a purity of >99.9% with undetectable T cell contamination based on both flow cytometry and CD3 mRNA evaluations [15], [17], [20].

Purified monocytes from HIV-1 infected patients were allowed to differentiate for 3 d in Iscove’s medium (IMDM; Invitrogen, Carlsbad, CA) complete media containing 10% heat-activated AB+ human serum (Valley Biomedical, Winchester, VA), 1000 U/ml recombinant human macrophage colony-stimulating factor (rhMCSF; R&D Systems, Minneapolis, MN) and 1× penicillin/streptomycin (pen/strep; Invitrogen). Meanwhile, PBMC from healthy donors (HIV, HBV and STD negative; Puget Sound Blood Center, Seattle, WA) were subjected to CD8+ cell depletion using magnetic bead-conjugated anti-human CD8 Ab (Dynal, Oslo, Norway) and cultured in RPMI media (Invitrogen) supplemented with 15% fetal bovine serum (FBS) (Invitrogen), 1× pen/strep and 1 µg/ml phytohaemagglutinin (PHA; Remel Products, Lenexa, KS) for 2–3 d.

After 3 d of differentiation, patient MDM were co-cultivated with PHA stimulated and CD8-depleted donor PBMC in IMDM complete medium with no MCSF supplement. Co-cultures were maintained for 4 w with the addition of fresh PHA-stimulated, CD8-depleted donor PBMC every 3 d. The culture supernatants were collected every 3–4 d, and viral replication was monitored using an HIV-1 p24^CA^ antigen capture assay kit (National Cancer Institute, Frederick, MD) following the manufacturer’s instructions. HIV-1 primary isolate expansions were titrated by Reed-Muench 50% tissue culture infectious dose (TCID_50_) assay.

### Isolation of Infectious HIV-1 from Patient CD4^+^ T cells

Patient CD4^+^ T cells were purified from PBMC with a similar two-step strategy to that used for monocyte isolation. First, CD4^+^ T cells from study subjects were enriched using the CD4^+^ T cell Isolation Kit II (Miltenyi) according to the manufacturer’s instructions. Second, a positive selection using microbead-conjugated anti-human CD4 Ab (Miltenyi) was performed on the enriched CD4^+^ T cells. Purified patient CD4^+^ T cells were cultured in RPMI 1640 complete media supplemented with 100 U/ml IL-2 for 24 h, followed by co-cultivating with PHA-stimulated and CD8-depleted donor PBMC (donor PBMC to patient CD4^+^ T cell ratio of 2∶1). Co-cultures were maintained for 1–4 w with the addition of fresh PHA-stimulated CD8-depleted donor PBMC every 3–4 d [45]. The culture supernatants were collected every 3–4 d, and viral replication was monitored using the HIV-1 p24^CA^ antigen capture assay kit (NCI). The isolates were titrated by Reed and Muench TCID_50_ assay.

### Co-receptor Usage Prediction

As positive or negative controls to validate indicator cell lines, HIV-1 laboratory-adapted strains including BaL, IIIB, ADA and SF2 were obtained from NIH AIDS Research and Reference Reagent Program; in addition, a known R5-tropic HIV-1 primary isolate acquired by our lab, 1192PB was used as a control virus. Co-receptor usage was predicted according to the V3 sequences of different isolates by employing the 11/25 rule [86], Geno2pheno (http://coreceptor.bioinf.mpi-inf.mpg.de/index.php) [58] with a false-positive rate of 10%, and the Position-Specific Scoring Matrix (PSSM) approach (http://ubik.microbiol.washington.edu/computing/pssm/) [59].

### Biological Confirmation of the Co-receptor Usage

Three U87 cell lines (NIH AIDS Research and Reference Reagent Program) were tested, including U87.CD4, U87.CD4.CXCR4 and U87.CD4.CCR5. Cells were thawed and passaged once before being seeded into 24-well plates at 1×10^4^ cells/well, and incubated 6 h in complete DMEM containing 300 µg/ml G418 (Invitrogen). An additional 1 µg/ml of puromycin (Sigma-Aldrich, St Louis, MO) was supplemented in both U87.CD4.CXCR4 and U87.CD4.CCR5 media, but not in U87.CD4 media. Cells were infected with HIV-1 at MOI = 0.01 and HIV-1 p24 antigen production was monitored.

Three U373-MAGI (MAGI) cell lines (NIH AIDS Research and Reference Reagent Program) were tested, including MAGI.CD4, MAGI.CD4.CXCR4 and MAGI.CD4.CCR5. Cells were cultured in complete DMEM containing 0.2 mg/ml G418 and 0.1 mg/ml hygromycin B (Invitrogen), and MAGI.CD4.CXCR4 and MAGI.CD4.CCR5 cell cultures were additionally supplemented with 1.0 µg/ml puromycin. Cells were infected with HIV-1 at MOI = 0.01 and 5-bromo-4-chloro-3-indoyl-β-D-galactoside (X-gal; Sigma) staining was performed. Cells were observed using a Zeiss microscope, and images were acquired using the AxioVision software (Carl Zeiss, Jena, Germany).

### Cyto-immunochemistry

The immune-detection of target molecules was performed as described previously [33]. Briefly, MDM were allowed to attach on cover glass while infection experiment performed. While staining, primary Abs used included mouse anti-human CD14 Ab, mouse-anti-HIV-1 p24 Ab (Dako, Carpenteria, CA); secondary fluorescence-conjugated Abs and DAPI-containing mounting solution were obtained from Invitrogen. Samples were observed with a Zeiss LSM 510 META NLO microscope (Zeiss, Thornwood, NY).

### HIV-1 PCR and Sequencing

PCR primers and conditions for *env* C2-V3-C3 were described previously [15], [17], [20]. HIV-1 *gag*, *polA* (5′ half of the *pol* region) and *polB* (3′ half of the *pol* region), was also amplified from viral cDNA with nested PCR. Primers were designed according to the HIV-1 HXB2 sequence from HIV-1 Database (Los Alamos National Laboratories, Los Alamos, NM); they were: outer primers including PG1 (positions 790-814; 5′-atgggtgcgagagcgtcggtattaa-3′) and PG4 (positions 2408-2382; 5′- acctccaattccccctatcatttttgg-3′), as well as inner primers including PG7 (positions 880-902; 5′-ctaaaacatatagtatgggcaag-3′) and PG2 (positions 2357-2328; 5′- ttcttctaatactgtatcatctgctcctgt-3′). Primers for *polA* were: outer primers of POL3 (positions 2003–2029; 5′-gcagggcccctaggaaaaagggctgtt-3′) and P58 (positions 3800-3777; 5′-gacaaactcccactcaggaatcca-3′), as well as inner primers of POL1 (positions 2031–2057; 5′-gaaatgtggaaaggaaggacaccaaat-3′) and P56 (positions 3758-3725; 5′-tgtccaccatgcttcccatgtttccttttgtatg-3′). Primers for *polB* were: outer primers of POL9 (positions 3425–3450; 5′-aataccactaacagaagaagcagagc-3′) and POL8 (5350-5325; 5′-ctgctaggtcagggtctacttgtgtg-3′), as well as inner primers of POL7 (positions 3528–3553; 5′-gcagaaatacagaagcaggggcaagg-3′) and POL6 (positions 5220-5195; 5′-ccctagtgggatgtgtacttctgaac-3′). HIV copy numbers were calculated based on limiting-dilution PCR with the QUALITY program as previously described [61]. HIV-1 sequences diversity was calculated using the HKY85 evolutionary model with the program DIVEIN [87].

### Statistical Analysis

Data were analyzed for statistical significance by two-tailed Student’s *t*-test, one-way or two-way ANOVA with Student *post hoc* test using GraphPad Prism version 5.02 (GraphPad Software, Inc., San Diego, CA), or by GEE analysis by employing the “pwr” package for R (http://cran.r-project.org/web/packages/pwr/index.html). *P*<0.05 was deemed significant.
